# Comparative Transcriptional Analysis Identified Characteristic Genes and Patterns in HIV-Infected Immunological Non-Responders

**DOI:** 10.3389/fimmu.2022.807890

**Published:** 2022-01-28

**Authors:** Xiaosheng Liu, Ling Lin, Lianfeng Lu, Xiaodi Li, Yang Han, Zhifeng Qiu, Xiaoxia Li, Yanling Li, Xiaojing Song, Wei Cao, Taisheng Li

**Affiliations:** ^1^ Tsinghua-Peking Center for Life Sciences, Beijing, China; ^2^ Department of Basic Medical Sciences, School of Medicine, Tsinghua University, Beijing, China; ^3^ Department of Infectious Diseases, Peking Union Medical College Hospital, Peking Union Medical College and Chinese Academy of Medical Sciences, Beijing, China

**Keywords:** HIV, INR, CD4 recovery, IFI27, IFN response pathway

## Abstract

**Purpose:**

The incomplete immune reconstitution is a complex phenomenon among human immunodeficiency virus (HIV)-infected patients despite the fact that they have achieved persistent viral suppression under the combined antiretroviral therapy. This study aims to screen and verify the immunological characteristics and underlying mechanisms of immunological non-responders (INRs).

**Methods:**

The RNA-seq and the differentially expressed genes (DEGs) analysis were used to explore potential characteristics among INRs. Gene Ontology (GO) enrichment, ingenuity pathway analysis (IPA) analysis, Gene set enrichment analysis (GSEA) analysis, and the weighted gene co-expression network analysis (WGCNA) were used to explore the potential mechanism. The transcriptional meta-analysis was used to analyze the external efficiency.

**Results:**

The RNA-seq identified 316 DEGs among INRs. The interferon signaling pathway was enriched *via* GO and IPA analysis among DEGs. The combined GSEA and WGCNA analysis confirmed that the IFN response was more correlated with INR. Furthermore, IFI27 (IFN-α Inducible Protein 27, also known as ISG12) was chosen based on combined DEG analysis, WGCNA analysis, and the transcriptional meta-analysis conducted on other published datasets about INRs. The expression of IFI27 was significantly negatively correlated with the CD4+ T-cell counts of PLWH, and the predictive efficiency of IFI27 level in distinguishing PLWH with poor immune recovery was also with significant power (AUC = 0.848).

**Conclusion:**

The enhanced expression of IFI27 and the IFN response pathway are among the important immunological characteristics of INRs and exhibited promising efficiency as biomarkers for CD4^+^ T-cell recovery.

## Introduction

The hallmark of the acquired immunodeficiency syndrome (AIDS), caused by the human immunodeficiency virus (HIV) infection, is accompanied by continued HIV replication and profound decline of circulating CD4^+^ T lymphocyte cells (1). Nevertheless, approximately 10%–40% of people living with HIV/AIDS (PLWH) are unable to achieve optimal CD4^+^ T-cell recovery despite virological suppression under the combined antiretroviral therapy (cART) (2). The U.S. Department of Health and Human Services (DHHS) considered that PLWH with CD4^+^ T cells less than 350–500 cells/μL after 4–7 years of suppressive cART therapy as immunological non-responders (INRs) (2, 3). INRs have increased risks of mortality and AIDS-related and non-AIDS-related events than the immunological responders (IRs), making it essential to explore the relevant mechanisms and develop therapeutic interventions (2, 4).

The precise number of peripheral CD4^+^ T cells results from the balance of production, destruction, and traffic between peripheral blood and lymphatic tissue. Based on the current knowledge, there are two primary causes: the declined production caused by thymic dysfunction and the enhanced destruction caused by immune activation (IA), which lead to the development of incomplete immune reconstitution (2, 5, 6). The persistence of chronic immune activation (IA) is considered as one of the best valued immunological features of INRs (7–9). The association of residual systemic inflammation with clinical outcomes among HIV/AIDS patients has been noted and continues to be evaluated, especially among INRs (10, 11). Given the lack of efficient thymic-improving therapy, along with the correlation between abnormal immune activation and adverse clinical outcomes, several clinical attempts had been made to reduce the chronic IA for years, however, with limited satisfied results.

To identify a relatively effective clinical intervention in treating INRs, a more profound understanding of underlying mechanisms for the persistent CD4 deficiency and chronic IA remains imperative (12). Several factors have been proposed, including the residual HIV replication, co-infection, microbial translocation, intestinal flora, and the imbalance of Treg cells, Th17 cells, etc. to explain the chronic IA among INRs (10, 13–22). These factors are considered to induce the activation of inflammatory pathways, such as interferon (IFN), NF-κB, caspase, and TLR signaling pathways, which would boost the enhanced secretion of downstream inflammatory factors, and eventually lead to the dysfunction and destruction of CD4^+^ T cells (2, 12, 20, 23–29). Among these various causes and immunological pathways, searching for the most prominent characteristic genes and patterns of chronic IA would help to understand the precise mechanisms of INRs.

Currently, the whole transcriptomic profiling is an unbiased approach to studying the gene expression patterns among tissues or cells, and it has been involved as the critical method of disease mechanisms discovery (30). To better understand the characteristics of gene expression in INRs and IRs, we conducted RNA-seq on INR patients and analyzed with comparative methods, including the ingenuity pathway analysis (IPA), the gene set enrichment analysis (GSEA), and the weighted gene co-expression network analysis (WGCNA) (31–33). To improve the external efficiency, we additionally conducted the meta-transcriptional analysis on published gene expression datasets of INRs and validated the identified genes (34, 35). The combined approaches have been involved in the mechanism discovery of several diseases, such as chronic obstructive pulmonary disease (36), multiple sclerosis (37), Alzheimer’s disease (38), and cancer (39). Up to now, our study is the first to use comparative transcriptional analysis to explore the gene signature of peripheral blood related to INR. These comprehensive bioinformatic analyses aim to provide underlying mechanisms of chronic IA in INR and information for subsequent research and personalized treatment.

## Materials and Methods

### Participants and Selection Criteria

The patients were recruited from the HIV/AIDS clinics of the Department of Infectious Disease in Peking Union Medical College Hospital (PUMCH). These patients received cART and were followed regularly. INR was defined by those patients who (1) were HIV seropositive (2); were treated with cART for more than 4 years (3); achieved virologic control (VL < 20 copies/mL) for more than 3.5 years; and (4) continued low CD4^+^ T-cell counts (<350 cells/μL). The IR definitions were the same as INR, except they achieved optimal CD4^+^ T-cell recovery (>500 cells/μL) in at least two consecutive measurements. The demographic information, immunological characteristics, co-infection, and treatment regimens were collected and compared.

### Lymphocyte Subsets and HIV-1 Viral Measurement

EDTA-anticoagulated fresh whole blood of patients was collected and incubated with monoclonal fluorescence antibodies, and the percentage of immune cells within lymphocytes was analyzed by a flow cytometer (LSR Fortessa, BD Biosciences), including T cells (CD45^+^CD3^+^), B cells (CD45^+^CD3^-^CD19^+^), NK cells (CD45^+^CD3^-^CD16^+^CD56^+^), CD4^+^ T cells (CD45^+^CD3^+^CD4^+^), CD8^+^ T cells (CD45^+^CD3^+^CD8^+^), naïve CD4^+^ T cells (CD45^+^CD3^+^CD4^+^CD62L^+^CD45RA^+^), and RTE (recent thymic emigrant) CD4^+^ T cells (CD45^+^CD3^+^CD4^+^CD45RA^+^CD31^+^). The absolute immune cell number was converted from multiplying immune cell percentage and the lymphocyte counts obtained from routine blood tests. The plasma of patients was isolated from whole blood through centrifugation to measure the plasma HIV-1 RNA (plasma viral load) to monitor the efficiency of cART. According to the manufacturer’s recommendations, the viral load measurement was determined by the COBAS AmpliPrep/COBAS TaqMan V2.0 RT-PCR (Roche). The lower limit of HIV-1 viral load is 20 copies/mL.

### Transcriptome Analysis With RNA-seq

The PBMC of patients was isolated from whole blood with density gradient centrifugation using separation medium (Ficoll-Paque PLUS, GM) and washed in PBS (Beijing Solarbio Science & Technology Co., China). Total RNA was extracted using TRIzol (Invitrogen Life Technologies, USA) and RNeasy Mini Kit (Qiagen company, GM). After extraction, the RNA purity was determined by a NanoPhotometer spectrophotometer (IMPLEN, CA, USA), the concentration was determined by a Qubit 2.0 Fluorometer (Life Technologies, CA, USA), and the integrity was determined by an RNA Nano 6000 Assay Kit in the Agilent Bioanalyzer 2100 system (Agilent Technologies, CA, USA) from the Biomark Technologies company (Qingdao, China).

The transcriptome sequencing on extracted total RNA was conducted in Hiseq 2500 platform (Illumina, San Diego, CA, USA) and generated with paired-end reads. The adaptor sequences and low-quality raw reads were removed as quality control and transformed into a clean read. After data processing, the clean reads were mapped to the reference genome using TopHat2 software, and mapped reads would be annotated and further analyzed as detectable genes (40).

### Differential Expression and Enrichment Analysis

The RNA-seq data were analyzed by DESeq2 R package to identify differentially expressed genes (DEGs) between different disease conditions. The *p*-values were adjusted using Benjamini and Hochberg’s approach for controlling the false discovery rate. Genes with an adjusted *p*-value <0.05 and fold change (FC) value > |1.5| (equals log2FC > |0.584|) between groups were concluded as DEGs.

The canonical pathway enrichment analysis on the above DEGs was performed on IPA software (Ingenuity Systems; Qiagen China Co., Ltd.). *Z*-score was calculated to infer the bioactivated status of relative pathways, and those canonical pathways with *p*-value < 0.001 (equals -LogP > 3.0) and *Z*-score > |2.0| were considered as significantly activated (*Z*-score > 2.0) or significantly inhibited (*Z*-score < −2.0) pathway.

### Gene Set Enrichment Analysis

The Gene Set Enrichment Analysis (GSEA) on whole-genome expression was performed on the GSEA software (v4.1.0). The hallmark gene set was downloaded from MSigDB Collections and considered the reference genome (41). The enrichment scores (ES) were calculated from weighted Kolmogorov–Smirnov-like statistics, and its magnitude reflected the correlation between a gene set and phenotype. The higher ES of the gene set means the higher possibility that this gene set was expected enriched in a particular phenotype.

### Weighted Gene Co-Expression Network Analysis

The weighted gene co-expression network analysis (WGCNA) was performed on the SangerBox platform (http://sangerbox.com/Tool) with three steps, and default settings were used as thresholds. WGCNA can analyze the relationship between genes and divide them into multiple modules. The hub genes were defined as a high degree of connectivity (>0.90) inside different modules and significantly (adjusted *p*-value < 0.05) correlated to phenotype.

### Transcriptional Meta-Analysis

For thorough searching of public datasets, the Preferred Reporting Items for Systematic Reviews and Meta-Analyses (PRISMA) workflow chart was made according to recommendations (42). The selected datasets were further input to ImaGEO to conduct the transcriptional meta-analysis (43). Since the divergence of included datasets, the effect size with the random-effect model was used as the estimation model. The meta-DEGs were defined as genes with adjusted *p*-value < 0.05, Qpval > 0.05, and tau^2^ = 0 in the output results.

### HIV-1 Cell-Associated DNA and RNA Measurement

The total cellular DNA and RNA were extracted from isolated PBMC samples by QIAamp DNA Mini Kit (Qiagen, Valencia, California) and HiPure Total RNA Plus Mini Kit (Magen, Guangzhou, China), respectively, and then amplified and quantified with the HIV DNA and cell-associated RNA (ca-RNA) quantitative detection kits (SUPBIO, Guangzhou, China) on the Roche LightCycler 480 (LC480) real-time PCR platform. The quantification of HIV caDNA and caRNA was multiplied by the percentage of CD4^+^ T cells in PBMC and the quantification of DNA/RNA copies among 1× 10^6^ cells.

### Statistical Analyses

For continuous variables with normal distribution, data were presented as mean and standard deviations and were analyzed by Student’s *t*-test. For variables with non-normal distribution, data were expressed as median and range of IQR, and the *p-*values were determined by an unpaired, two-tailed Mann–Whitney test. The correlations were analyzed using the Spearman test. Correlations were analyzed using the Spearman test. The *p*-value < 0.05 was considered statistically significant. All statistical analyses were performed using SPSS (version 25.0, SPSS Inc., Chicago, IL, USA) and GraphPad Prism (v8.0.1, GraphPad Software, La Jolla, CA).

## Results

### Characteristics of the Patients

We identified 421 PLWH treated with cART for more than 4 years in the HIV/AIDS clinical center of PUMCH, and 58 patients (20 INRs and 38 IRs) were included eventually based on the criteria described in the methods. The median age of the included patients was 46 (IQR, 36–53) years old, and 89.7% were male. The INRs experienced poor immune reconstitution and had a significantly lower CD4^+^ T-cell count of median 252 (IQR, 211–290) cells/μL when compared to IRs [900 (IQR, 829–1,116) cells/μL, *p* < 0.0001], despite the similar duration of suppressed cART treatment (**Table 1**).

**Table 1:** Clinical characteristics of enrolled 58 HIV/AIDS patients.

Patient characteristics	INR group (*n* = 20)	IR group (*n* = 38)	*p*-value
**Demographics**			
Age, years	46 (40–57)	44 (34–52)	0.223
Sex, male	18 (90%)	34 (89%)	0.951
**Follow-up**			
Treatment time, years	6 (4–8)	7 (6–10)	0.128
**Immunological characteristics**			
Lymphocyte, 10^9^/L	1.47 (1.34–1.95)	2.56 (2.24–2.99)	<0.001
T-cell counts, 10^9^/L	1.11 (0.82–1.24)	1.83 (1.63–2.12)	<0.001
B cell counts, 10^9^/L	0.18 (0.10–0.24)	0.24 (0.18–0.33)	0.004
NK cell counts, 10^9^/L	0.34 (0.15–0.54)	0.37 (0.19–0.58)	0.852
CD4^+^ T-cell counts,/μL	252 (211–290)	900 (829–1116)	<0.001
Naïve CD4^+^ T-cell counts,/μL	53 (43–68)	358 (246–558)	<0.001
RTE CD4^+^ T-cell counts,/μL	13 (6–22)	114 (68–161)	<0.001
CD8^+^ T-cell counts,/μL	682 (540–852)	789 (559–886)	0.518
CD4/CD8 ratio	0.4 (0.3–0.5)	1.3 (1.0–1.7)	<0.001
**Baseline characteristics**			
Nadir CD4^+^ T-cell counts,/μL	25 (12–98)	328 (118–474)	<0.001
Naïve CD4^+^ T-cell counts,/μL	3 (1–12)	111 (22–164)	<0.001
Viral load, log10 cps/mL	5.1 (4.7–5.3)	4.8 (4.3–5.2)	0.288
**cART Treatment**			
NNRTI based	16 (80%)	31 (82%)	0.885
INSTI based	2 (10%)	3 (8%)	0.788
PI based	2 (10%)	4 (10%)	0.951
**Previous co-infection**			
HBV	8 (40%)	17 (45%)	0.731
HCV	2 (10%)	0 (0%)	0.049
CMV	12 (60%)	26 (68%)	0.525

RTE, recent thymic emigrant; NNRTI, non-nucleoside reverse transcriptase inhibitor; INSTI, integrase strand transfer inhibitor; PI, protease inhibitor; HBV, hepatitis B virus; HCV, hepatitis C virus; CMV, cytomegalovirus.

**Figure 1:**
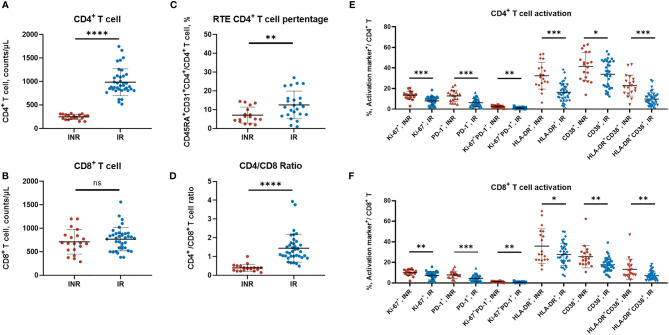
The T-cell subtypes and activation profiles of HIV/AIDS patients. **(A–D)** The CD4^+^ T-cell counts, CD8^+^ T-cell counts, RTE CD4^+^ T-cell percentage, and the CD4/CD8 ratio between INR and IR patients. Each dot represents one patient. **(A, B, D)** contained 20 INR and 38 IR samples, **(C)** contained 17 INR and 24 IR samples. **(E, F)** The CD4^+^ T and CD8^+^ T-cell activation profiles were measured by flow cytometry (surface markers including HLA-DR, CD38, PD-1, and intracellular marker Ki67). Data are mean ± SD, and the *p*-values were determined by a two-tailed Mann–Whitney test. *p < 0.05 **p < 0.01, ***p < 0.001, ****p < 0.0001; NS, not significant.

These included INRs showed lower naïve CD4^+^ T-cell counts (*p* < 0.001), lower RTE CD4^+^ T-cell counts (*p*< 0.001), and lower CD4/CD8 ratio (*p* < 0.001) when compared with IRs (**Table 1**). Meanwhile, the INRs exhibit elevated expression levels of HLA-DR, CD38, Ki-67, and PD-1 on CD4^+^ T cells and CD8^+^ T cells (**Figure 1**), showing the enhanced immune activation and exhaustion in the INRs. In line with our previous understanding, the insufficient thymic output and the elevated immune activation were composited as the salient immunological characteristics of INRs.

### RNA-seq and DEGs Analysis

For a better understanding of the immune activation profiles of INR patients, we conducted the RNA-seq on 16 patients (8 INRs and 8 matched IRs) to evaluate the differential gene expression profiles. There was no significant statistical difference in sex (*p*= 1.000), age (*p*= 0.959), duration of cART (*p*= 0.853), and the nadir CD4^+^ T cells [INRs vs. IRs: 12 (IQR, 6–42) vs. 34 (IQR, 8–72) cells/μL (*p*= 0.505)] between the selected and matched patients, except the current CD4^+^ T cells [INRs vs. IRs: 223 (IQR, 166–250) vs. 756 (IQR, 658–921) cells/μL (*p* < 0.001)] (see **Table S1**).

**Figure 2:**
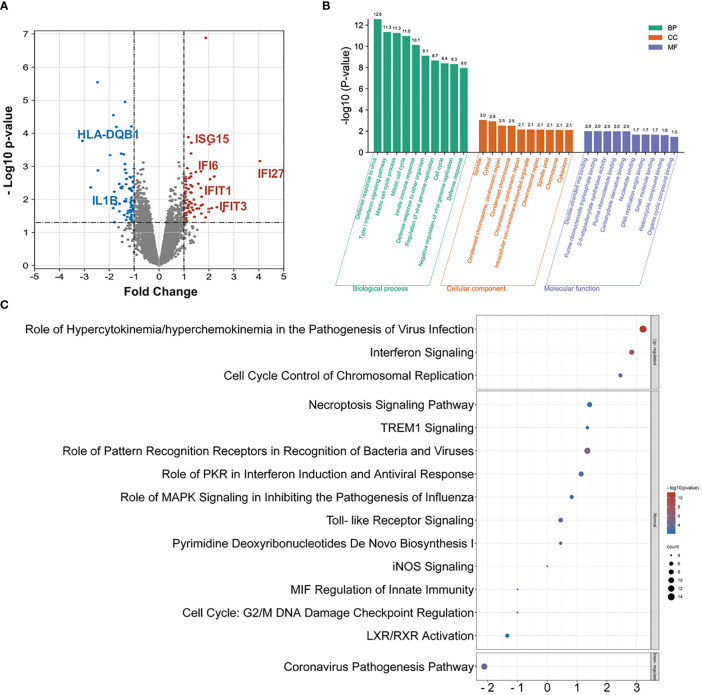
The RNA-seq and DEG analysis among INR and IR groups. **(A)** The volcano chart on differentially expressed genes (DEGs) identified by RNA-seq between INR and IR patients (DEG defined as *p*-value < 0.05 and FC > |1.5|). **(B)** The GO analysis on upregulated DEGs and only the top 10 enriched terms in each category were shown. **(C)** Top enriched canonical pathways following ingenuity pathway analysis (IPA). Canonical pathways with a *p*-value < 0.001 were labeled, and the *x*-axis represents the value of the *Z*-score.

The PBMC samples were collected and analyzed with RNA-seq. A total of 316 DEGs are identified, namely, 146 upregulated genes and the remaining downregulated 170 genes in the INRs compared to IRs (**Figure 2A** and **Table S2**). The Gene Ontology (GO) functional classification analysis identified 99 biological processes groups (BP), 18 cellular component groups (CC), and 11 molecular function groups (MF) among 146 up-DEGs. In contrast, no significant enriched group was identified among 170 down-DEGs (see **Table S3**). The defense response to virus (GO:0051607), the type I interferon signaling pathway (GO:0060337), and the mitotic cell cycle process (GO:1903047) were the most highly represented terms in BP (**Figure 2B**).

The IPA was further performed to evaluate the function of the assembled up-DEGs and down-DEGs. A total of 54 pathways were enriched with predicted *Z*-scores, and 15 of them were considered statistically significant (see **Table S4a**). Among these, only the role of hypercytokinemia or hyperchemokinemia in the pathogenesis of virus infection pathway (ratio = 0.175), the interferon signaling pathway (ratio = 0.222), and the cell cycle control of chromosomal replication pathway (ratio = 0.107) were considered upregulated (*Z*-score > 2) and the coronavirus pathogenesis pathway (ratio = 0.057) was considered downregulated (*Z*-score < −2) (**Figure 2C**). Notably, the interferon signaling pathway exhibited the highest impacted ratio among the four regulated pathways. The upstream regulator analysis also identified a robust activation of the IFN-associated regulators among these DEGs (see **Table S4b**).

### Gene Set Enrichment Analysis

**Figure 3:**
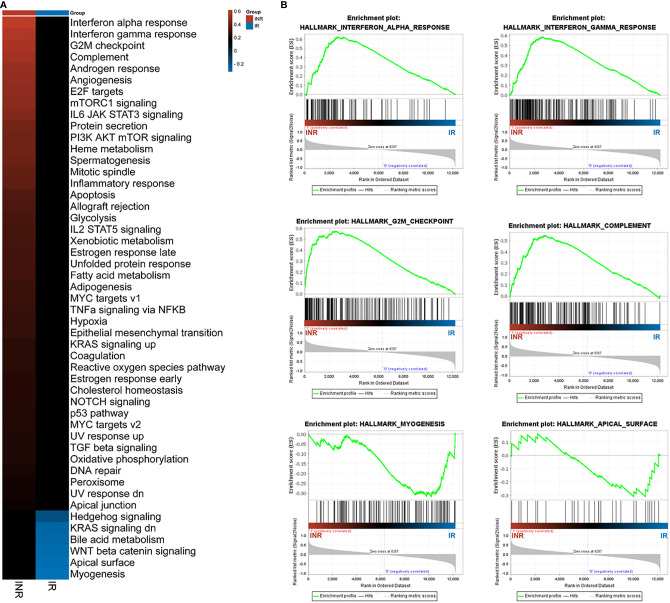
The GSEA analysis results. **(A)** The GSEA analysis results among patients. The colored patches were quantified with enrichment score (ES) from GSEA results. **(B)** The enriched top 4 pathways in INRs and the top 2 pathways in IRs. The *y*-axis represents ES, and the *x*-axis denotes genes (vertical black lines) represented in gene sets. The colored band at the bottom represents the degree of correlation of genes with the INR phenotype (red for positive and blue for negative correlation).

The former GO and IPA analysis mainly focused on the identified 316 DEGs; we conducted the gene set enrichment analysis (GSEA) on whole-genome data to further illustrate the holonomic biological functions of the transcriptome. With 12,195 entries of mRNA expression data, the GSEA analysis identified 6,287 (51.6%) upregulated genes in INRs and 5,908 (49.7%) upregulated in IRs. The hallmark pathways mapping showed 43 enriched pathways in INRs and 6 enriched pathways in IRs (**Figure 3A**). Among these enriched pathways, the IFN-α response (ES = 0.62, signal%= 85%) and the IFN-γ response (ES = 0.59, signal%= 69%) were the top gene sets with the highest enrichment score in the INR group (**Figure 3B**). Forty-eight core genes were commonly enriched in both the IFN-α response and the IFN-γ response pathway, and 31 of 48 (64.6%) were not included in the former 316 DEGs, illustrating the additional functional role of non-DEGs (see **Table S5**).

### Weighted Gene Co-Expression Network Analysis

**Figure 4:**
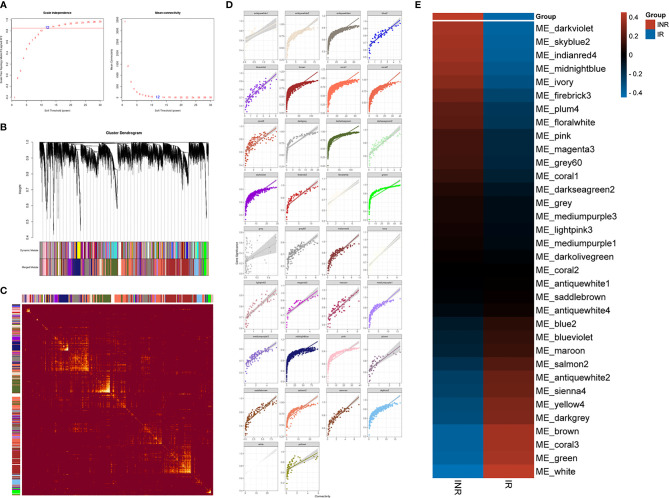
The WGCNA analysis results. **(A)** The analysis of the scale-free fit index (left) and the mean connectivity (right) to determine the soft-thresholding powers. **(B)** Cluster dendrogram of genes modules, with gene dissimilarity based on the topological overlap, together with assigned 34 module colors. **(C)** The network heatmap plot among 34 modules and the progressively saturated yellow colors indicated higher overlap between modules. **(D)** Scatterplot of the intramodular analysis (module membership versus gene significance) of genes in every separate module. **(E)** Module–trait relationship to the INR and IR phenotype. Each row responds to a colored module eigengene (ME) detected by WGCNA analysis and each column to a trait. The table is color-coded according to gene module and phenotype correlation coefficient, ranging from −1 to 1 (blue to red).

We additionally conducted the weighted gene co-expression network analysis (WGCNA) to explore the co-expression relationships among whole-genome data. With the correlation coefficient threshold selected as 0.85, the soft-thresholding power was set as 12 (**Figure 4A**). The WGCNA analysis identified 34 gene co-expression modules with internal relationships, and each of these was independent of other modules (**Figure 4B**). The brown module (*n* = 2007), the coral_1 module (*n* = 1366), the antique_white_4 module (*n* = 1272), the dark_olivegreen module (*n* = 1270), and the midnight_blue module (*n* = 1134) comprised the majority of 12,195 genes (**Figure 4C**). The weighted expression analysis showed good connectivity inside each gene module (**Figure 4D**). The module-trait correlation analysis showed that modules were related to INR, including the dark_violet module (*r* = 0.39), the skyblue_2 module (*r* = 0.38), the indian_red_4 module (*r* = 0.37), and the midnight_blue module (*r* = 0.35) (**Figure 4E**).

The midnight_blue module exhibited large gene counts (*n* = 1,134) and the higher correlation index (*r* = 0.35) with INR (see **Table S6**). The functional annotation showed that the type I interferon signaling pathway (GO:0060337) was the most significant progress (strength = 1.47, FDR = 7.12e-08) among 67 enriched biological progresses in the midnight_blue module (see **Table S7**). Several genes in the midnight_blue module such as IFI27 (*R* = 0.94), OAS1 (*R* = 0.93), STAT2 (*R* = 0.92), BST2 (*R* = 0.92), OAS2 (*R* = 0.92), XAF1 (*R* = 0.91), IFI35 (*R* = 0.91), MYD88 (*R* = 0.91), and SAMHD1 (*R* = 0.90) had high gene significance with INR and thus considered as hub genes. In total, there were 1,045 identified hub genes among 33 gene modules through WGCNA analysis that were considered related with INR and for further validation (see **Table S8**).

### Transcriptional Meta-Analysis

**Figure 5:**
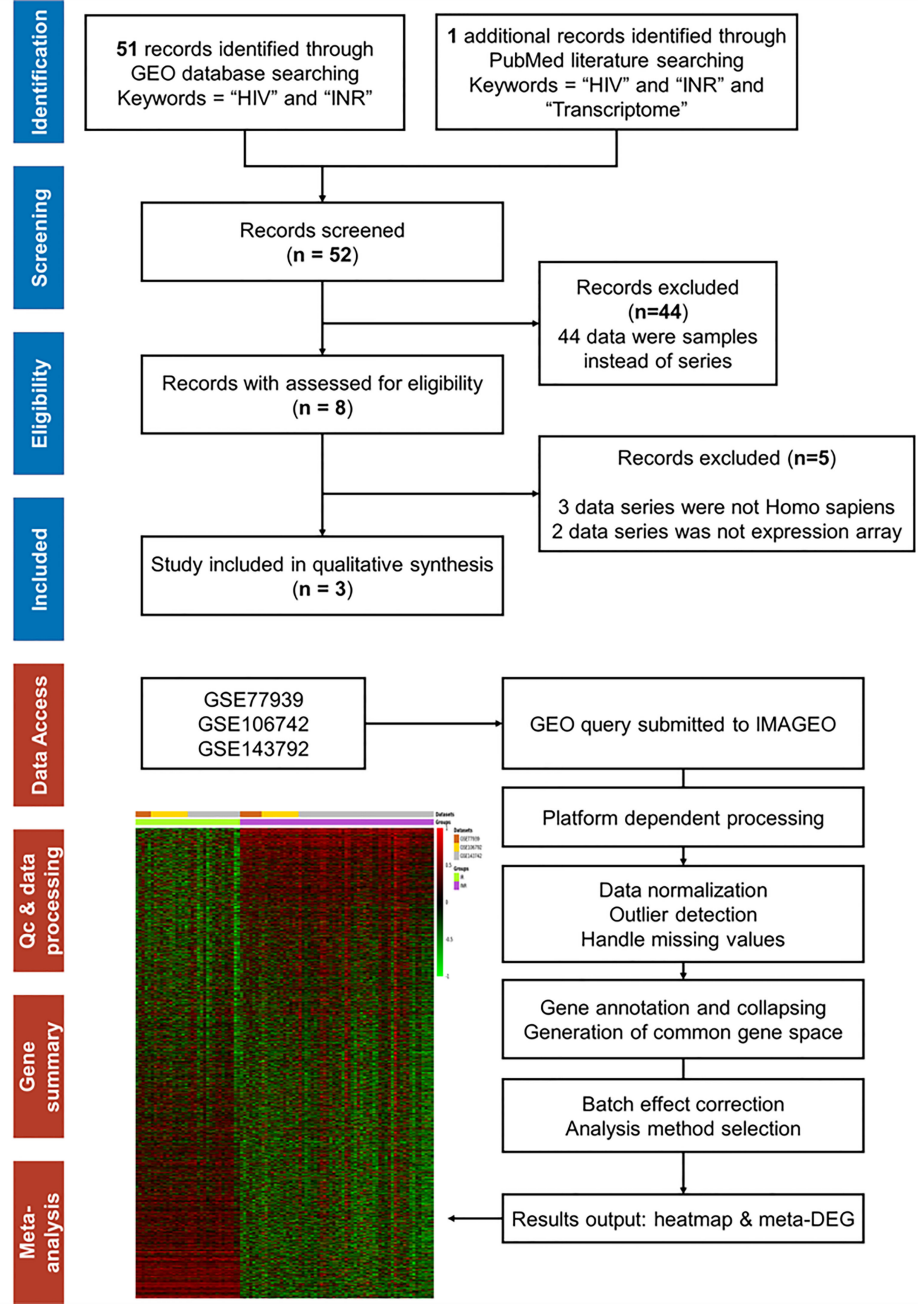
The PRISMA flow and search results for meta-analysis. The workflow diagram of database and literature search for mRNA expression array on INR patients and the selection process for inclusion in the meta-analysis. The workflow diagram of transcriptional meta-analysis on the IMAGEO website includes the following: First, input data were selected from public repositories. Secondly, the raw data were reprocessed to get gene expression matrices and quality controls were performed. Then, a common gene space for all the datasets was created where batch effect correction can be applied and, finally, the meta-analysis was performed. In our analysis, the Fisher *p*-value method [summary of −log(*p*-value) across studies] was selected as the analysis method. The allowed missing values (%) were defined as 10%, and the *p*-value < 0.05 was the adjusted *p*-value threshold.

To validate the external consistent of regulated genes, we further conducted the meta-analysis on published transcriptional data of INR patients. After screening 52 published records, we included three microarray datasets (GSE77939, GSE106792, and GSE143742) following the criteria of the PRISMA workflow (**Figure 5**).

In total, 63 INR and 34 IR samples from 3 datasets were included, and the dissimilarity in the immune recovery criteria, the microarray platform, and the cell types between different studies were undeniable (**Table 2**). Thus, the random effect test was chosen to reduce variance. After batching the expression baseline and examining the missing data, a total of 16,821 genes were included for further analysis, and the heatmap of whole-genome expression of 97 samples is shown in **Figure 5**. With the threshold of adjusted *p*-value < 0.05, Qpval > 0.05, and tau^2^ = 0 in the random effect test, 409 meta-DEGs (56 upregulated and 353 downregulated) were obtained (see **Table S9**).

**Table 2:** Clinical characteristics of enrolled 58 HIV/AIDS patients.

Datasets	INR	CD4	IR	CD4	VCT	Platform	Cell type
GSE77939	7	<250	5	>250	> 1	GPL15207	PBMC
GSE106792	12	<350	12	>500	> 2	GPL10558	CD71^+/-^ CD45RA^-^CD4^+^
GSE143742	44	<350	17	>500	> 3	GPL10558	CD4^+^ T cells
**Total**	**63**	–	**34**	–	–	–	–

VCT, virological control time, years.

### Validation and Efficacy Evaluation of Identified Genes

**Figure 6:**
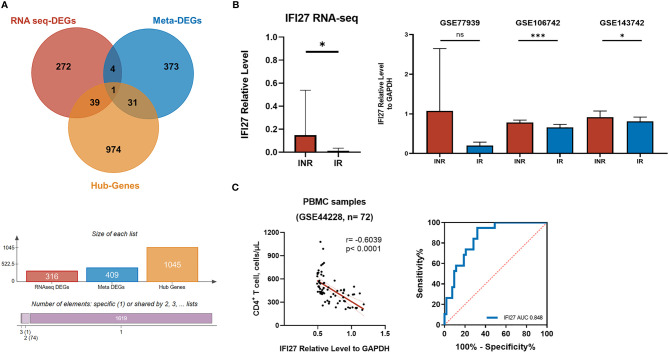
The screening of critical genes in the mechanism of INR. **(A)** The Venn diagram showing the overlapping of DEGs from our RNA-seq analysis (red), the meta DEGs identified from the transcriptional meta-analysis on published GEO data series (blue), and the hub genes from WGCNA analysis (yellow). **(B)** The relative expression level of IFI27 to GAPDH included transcriptome analysis. The probes of each GEO data series were transformed into gene symbols through R, and the geographical mean of the same gene was merged and calculated. The *p*-values of relative expression were determined by the random-effect model of IMAGEO. *p < 0.05, ***p < 0.001; NS, not significant. **(C)** The correlation between IFI27 expression and the CD4^+^ T-cell counts and the ROC curve.

Taken together, the IFI27 [IFN-α Inducible Protein 27, also known as IFN stimulated gene 12 (ISG12)] was the only gene that simultaneously identified as the DEG, the hub gene of WGCNA, and the meta-DEG (**Figure 6A**). The expression levels of IFI27 were consistently upregulated in INR patients across different datasets with different conditions, especially in the PBMC samples (**Figure 6B**). Furthermore, the expression of IFI27 in PBMC samples was significantly negatively correlated with the CD4^+^ T cells counts of PLWH, and the area under the curve (AUC) of the ROC curve was larger than 0.80 in dataset GSE44228 (**Figure 6C**). The sensitivity% and the specificity% were 94.7% and 67.9% as the relative expression levels of IFI27 to GAPDH > 0.60 in distinguishing PLWH with poor immune recovery (CD4^+^ T cells < 350 cells/μL) (see **Table S10**). These results supported the vital commonality of the enhanced ISG expression (especially IFI27) and the activated IFN signaling pathway in INRs.

### IFN Response and the Viral Response

**Figure 7:**
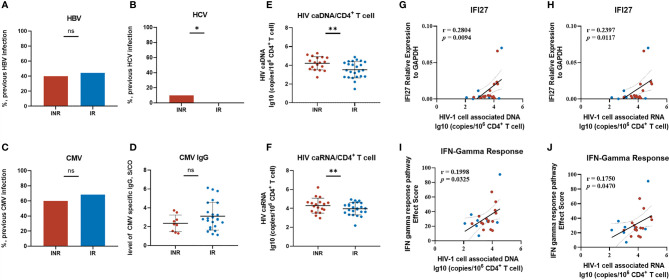
Exploring the driving factors of IFI27 expression and the IFN pathway. **(A–D)** The previously viral infection status of our INR and IR patients includes HBV, HCV, and CMV. **(E, F)** The level of HIV cell-associated DNA and RNA between groups. **(G–J)** The correlation of HIV caDNA/caRNA with IFI27 score and IFN-γ response score. The IFN response score was calculated by summing up the Effect Score (ES) in GSEA of every individual pathway-related gene. The *p*-values of infection rates among groups were determined by a two-tailed Mann–Whitney test. *p < 0.05, **p < 0.01; NS, not significant. The linear regression analysis was calculated by Spearman’s rank correlation coefficient when *p*-values < 0.05 were considered significant.

Although we identified the characteristic gene and patterns of INRs, questions remained to be answered as to what might be the driving factors of elevated IFI27 expression and the activated IFN response among these patients. The essential role of IFN signaling in responding to viral infection was well known. However, there was no significant difference in the rate of hepatitis B virus (HBV), cytomegalovirus (CMV) co-infection, or the level of CMV-specific IgG titer among our INR and IR patients (**Figures 7A, C, D** and **Table 1**). It was noted that the limited number of INR patients (2/20, 10%) were previously infected with hepatitis C virus (HCV), making it statistically different between two groups while unable to explain the extensive abnormal enhanced IFN response among INR patients (**Figure 7B**).

Attention came back to HIV. Despite the fact that all of our INR and IR patients had achieved viral control (HIV-1 viral load less than 20 copies/mL) for years, the HIV reservoirs persisted and could produce replication-competent viruses under cART. We measured the HIV-1 cell-associated DNA (caDNA) and cell-associated RNA (caRNA) among these patients. The level of HIV-1 total DNA/CD4^+^ T cells in INRs was higher than in the IRs (*p* < 0.001) (**Figure 7E**), and a similar result was also observed in HIV-1 caRNA/CD4^+^ T cells (*p* < 0.001) (**Figure 7F**). Further correlation analysis showed the expression of IFI27 and the IFN-γ response score were linearly dependent on the level of HIV-1 caDNA and HIV-1 caRNA (**Figures 7G–J**), indicating that the persistence and the transcription of HIV reservoirs might be in alliance with the activated IFN pathway in INR patients.

## Discussion

The accessibility and activity of cART help to boost the rapid viral suppression by stalling various steps of the HIV-1 life cycle, and most HIV/AIDS patients can experience immune reconstitution after receiving cART (1). However, approximately 10%–40% of PLWH cannot achieve optimal CD4^+^ T-cell counts despite virological suppression and termed as INRs (2, 3). The suboptimal CD4^+^ T-cell recovery has been proved to be associated with a substantial increase in the risk of mortality, and AIDS-related and non-AIDS-related morbidity, calling for the urgency of investigating the underlying mechanisms of INR and developing relevant interventions (2, 4). Previously, studies focused on the gene expression profiles of the PBMC isolated from HIV-1 subtype-C infected INRs (44) and of the CD4^+^ T cells isolated from INRs (10, 45); these results provided valued but un-unified information due to the dissimilarity in sequencing platforms, cell types, and the selection criteria of included patients. In the present study, we conducted the RNA-seq on PBMC of INRs and matched IRs, and we constructed the consequent comparative transcriptional analysis and meta-analysis on other published datasets that helped to present the new insights into the molecular mechanisms of INR.

Our results agreed that the INRs exhibited persistent abnormal immune activation and insufficient thymic output as the important immunological characteristics. To reduce the bias, the further RNA-seq on INRs and matched IRs with similar nadir CD4^+^ T cell and other demographic profiles identified a total of 316 DEGs (146 up-DEGs and 170 down-DEGs) among groups. The GO and IPA analysis showed that the interferon signaling pathway was enriched among DEGs. Expanding to the whole genome expression data, the GSEA analysis confirmed that the IFN-α response (ES = 0.62) and the IFN-γ response (ES = 0.59) were the top gene sets with the highest ES in the INRs. The type I interferon signaling pathway (GO:0060337) was also the most significant progress in the midnight_blue module in the WGCNA analysis, which was identified with large gene counts (*n* = 1,134) and the higher correlation index (*r* = 0.35) with INR, showing the signature of enhanced IFN response among our INR patients.

Altogether, our main results revealed that the activated IFN response pathway contributed to the significant immunological characteristics of immune activation profiles among INRs. The binary connection between IFN signaling and HIV infection caused much interest and debate in the field (46). It has been reported that the enhancement of IFN signaling during acute infection would boost the anti-viral effects; however, the persisting activation of IFN signaling also results in immune depletion and reservoir maintenance during chronic infection (46). Studies showed that the administration of anti-IFNAR antibodies to ART-suppressed HIV-infected humanized mice reduced immune activation and reservoir size (47, 48). HIV/AIDS patients with higher circulating IFN-γ were associated with poor CD4^+^ T-cell recovery (49). In addition, clinical evidence had shown that applying the adjuvant IFN therapy to HIV/HBV co-infected patients resulted in the transient declines of CD4^+^ T-cell counts (50, 51). Taken together, we considered that limiting the contribution of the IFN response pathway in the maintenance of HIV reservoirs among INRs could be thought to be an important target for clinical improvement (52).

In addition, we conducted the meta-analysis on three published datasets and obtained 409 meta-DEGs (56 up-meta-DEGs and 353 down-meta-DEGs). The IFI27 from the IFN response pathway was the only gene that simultaneously identified as the DEG, the hub gene of WGCNA, and the meta-DEG. Notably, the association between HIV infection and ISG, especially IFI27, had been noticed years ago. Previous studies reported the relative higher IFI27 expression in the CD4^+^ T cells of PLHW with lower CD4^+^ T-cell counts (27), and also higher in the monocytes of PLWH with high HIV-1 viral load (53). Nevertheless, the expression of IFI27 in the CD8^+^ T cells was not significantly different from patients (27), and it was even downregulated in primary macrophages after HIV infection (54), while until now, the role and the driving factor of IFI27 expression in PLWH remained to be explored.

Through the analysis on RNA extracted from the whole PBMC, rather than specific cell types, and the research through unsupervised transcriptomic profiling on whole genome, instead of focusing on ISG as in previous studies, our results helped to provide relative unbiased insights of enhanced IFI27 expression among INR patients. We further examined the relationship of IFI27 with CD4^+^ T-cell recovery, and the efficiency of IFI27 was validated in another dataset. We found that the expression of IFI27 was significantly negatively correlated with the CD4^+^ T cells counts of PLWH, and the relative expression level of IFI27 to GAPDH > 0.60 was shown with significant power in distinguishing PLWH with poor immune recovery (CD4^+^ T cells < 350 cells/μL). The validated results supported the vital commonality of the enhanced ISG expression (especially IFI27) and the activated IFN signaling pathway in INRs.

Moreover, we found that the expression of IFI27 and the IFN-γ response score were linearly dependent on the level of HIV-1 caDNA and HIV-1 caRNA. We speculated that the persistence and transcription of HIV reservoirs might be the driving factor of enhanced IFI27 expression and IFN response among INRs. Previous studies had shown the relationship between HIV reservoirs and suboptimal CD4 recovery (55–57). Interestingly, a recent study showed that the increased homeostatic cytokine levels, especially IL-7 and IL-15, were also associated with HIV reservoirs in T-cell subsets and poor immune reconstitution during cART (58). The role of proinflammatory and homeostatic cytokines in the connection between viral reservoirs recovery and the immunopathogenesis of chronic IA had been reported (59, 60). Of note, the HIV-1 Vpr protein was reported as an essential factor in the HIV-1 gene expression progress of cytokine-treated resting CD4^+^ T cells (61). The HIV-1 Vpr could be produced and released from HIV-1 sequestered tissue reservoirs during cART treatment (62, 63), and Vpr has been shown to participate in the HIV-mediated immune dysfunction and CD4^+^ T-cell depletion (64–69). Besides, the persistence of Vpr was able to upregulate the expression of various ISGs, including IFI27, in monocyte-derived dendritic cells (70). Hence, we hypothesize that the persistence of HIV reservoirs among INRs may induce the IFN response by the production of Vpr. However, the specific mechanisms underlying the interaction between proinflammatory and homeostatic cytokines and the HIV viral reservoirs remain further explored.

Although the present study is the first to conduct a comparative transcriptional analysis to explore the characteristic genes and patterns of INRs, our study also has limitations. Firstly, our transcriptional data were restricted to the limited matched PLWH from PUMCH; these patients were relatively young, and their CD4^+^ T-cell counts were mainly below 100/μL at cART initiation. Even though we had used the external datasets to validate the efficiency of identified genes, whether these deficiencies would affect the results or the conclusions needed a larger sample size to validate. Secondly, we applied the PBMC samples to measure HIV reservoirs, considering the availability and convenience of acquiring samples. Despite the fact that HIV reservoirs would be mainly represented in lymphoid tissues and gut-associated lymphoid tissue (GALT) (71, 72), our previous studies exhibited that the measurement of HIV reservoirs through peripheral blood also provided valuable information (73–76). Finally, we did not further study the connection between IFN response and microbial translocation, which was reported as a significant cause of persistent immune activation among INRs (20, 77–79). Recent studies had shown that the gut ISG levels were correlated with microbial translocation and microbial metabolites (80–82), while we were not powered to investigate the IFN gene expression profiles of the gut district and the data of gut microbiota had not been undertaken. Thus, the associations between IFN response and CD4 recovery and viral reservoirs observed in this study should be interpreted with caution. Nevertheless, our findings and conclusions could inform the further exploration of immunological characteristics and underlying mechanisms of INRs.

In conclusion, the present study reports the immunological gene and pattern from the comprehensive bioinformatic analysis of INR patients. Of note, our study finds and validated the involvement of the expression of IFI27 and the IFN response pathway in the immunological characteristics of INRs. The enhanced ISG expression and IFN response may align with the persistence and transcription of HIV reservoirs. Our group has registered and initiated several randomized clinical trials in treating INRs, and the coming results will further build our knowledge of the connection between HIV reservoirs, the IFN response, and the CD4^+^ T-cell recovery.

## Data Availability Statement

The original contributions presented in the study are deposited in the NCBI SRA repository, accession number PRJNA777889.

## Ethics Statement

The studies involving human participants were reviewed and approved by the institutional ethics board of Peking Union Medical College Hospital. The patients/participants provided their written informed consent to participate in this study.

## Author Contributions

XsL conducted the comparative transcriptional analysis, conducted the measurement of HIV reservoirs, and drafted the manuscript. LLi, LLu, XdL, XxL, YL, and XS provided regular clinic care on included patients. YH conducted the regular HIV-1 viral load measurement. ZQ conducted the regular cell cytometry measurement. WC reviewed the literature and revised the manuscript. TL advised the entire work. All authors reviewed the manuscript, provided feedback, and approved the manuscript in its final form.

## Funding

The research was supported by the national key technologies R&D program for the 13th five-year plan (2017ZX10202101).

## Conflict of Interest

The authors declare that the research was conducted in the absence of any commercial or financial relationships that could be construed as a potential conflict of interest.

## Publisher’s Note

All claims expressed in this article are solely those of the authors and do not necessarily represent those of their affiliated organizations, or those of the publisher, the editors and the reviewers. Any product that may be evaluated in this article, or claim that may be made by its manufacturer, is not guaranteed or endorsed by the publisher.
